# Chromosomal passenger complex-cyclin/CDK axis correlated with poor lung cancer prognosis

**DOI:** 10.7555/JBR.38.20240296

**Published:** 2025-02-08

**Authors:** Prerna Vats, Sakshi Nirmal, Ashok Kumar, Rajeev Nema

**Affiliations:** 1 Department of Biosciences, Manipal University Jaipur, Dehmi Kalan, Jaipur-Ajmer Expressway, Jaipur, Rajasthan 303007, India; 2 Department of Biochemistry, All India Institute of Medical Sciences (AIIMS), Bhopal, Saket Nagar, Bhopal, Madhya Pradesh 462020, India

Dear Editor,

Lung cancer is a major global health concern, with 2.2 million patients diagnosed in 2020. Non-small cell lung cancer (NSCLC) accounts for 80% of these cases, primarily comprising two subtypes: lung adenocarcinoma (LUAD) and squamous cell carcinoma (LUSC)^[1]^. Researchers use immunohistochemistry, next-generation sequencing, and single-cell RNA sequencing to study genetic alterations, tumor heterogeneity, and tumor microenvironments, aiming to identify potential therapeutic options for specific NSCLC subtypes^[2]^. Lung cancer is also the most prevalent cancer in India, representing 6.9% of new cases and 9.3% of all cancer-related deaths. The median overall survival for metastatic NSCLC patients is approximately four to five months, underscoring the need for novel therapeutic targets^[3]^. Several new gene expression targets are being investigated for cancer treatment, including chromosomal instability, which is often linked to defective cell cycles and regulatory checkpoints, such as cyclins/cyclin-dependent kinases (CDKs). During the cell cycle, cyclins and CDKs regulate four key checkpoints: G0/G1, S, G2, and M phase spindle assembly checkpoint. Abnormalities in these checkpoints may lead to abnormal chromosomal segregation and mitotic catastrophe, driving tumor aggressiveness. The chromosomal passenger complex (CPC), comprising Aurora kinase B (AURKB, also known as STK12), survivin (BIRC5), borealin (CDCA8), and INCENP, plays a crucial role in the M-phase spindle assembly checkpoint^[4]^.

The current study aimed to investigate the correlations between cell cycle checkpoints and the CPC, a key regulator of the cell cycle (metaphase), in LUAD. Increased levels of these components at the gene expression level in various cancers, including LUAD, are associated with early tumor recurrence and poor prognosis. Combining therapies targeting the CPC and cell cycle regulators may provide novel insights into the treatment of lung cancer. Treatment options for LUAD include surgery, chemotherapy, immunotherapy, radiotherapy, and targeted therapy. However, surgery is not suitable for tumors in advanced stages, chemotherapy may cause side effects, and targeted therapies are only effective for specific mutations^[5]^. Combination therapy targeting overexpressed genes may overcome resistance and improve treatment efficacy. By targeting multiple pathways simultaneously, this approach can prevent cancer cells from developing resistance to a single treatment and may allow for lower doses of each drug, potentially reducing side effects. Combination therapy targeting the CPC-cyclin/CDK axis may improve overall treatment efficacy and patient survival rates.

A preliminary study was carried out using the Kaplan–Meier Plotter (KM Plotter) database^[6]^, which provides access to the TCGA, GEO, and various microarray datasets. The KM Plotter is widely used for identifying the prognostic value of genes that may serve as predictive biomarkers. In the current study, we initially investigated the associations between the expression levels of CPC genes and three survival outcomes, including overall survival (OS), first progression survival (FPS), and post-progression survival (PPS), in lung cancer patients. As shown in ***Supplementary Table 1*** (available online) and ***Supplementary Fig. 1*** (available online), AURKB, a key component of the CPC, has the most significant effect on patient survival, with a hazard ratio (HR) of 1.78 for OS (95% CI, 1.58−2.01; *P* < 1.0 × 10^−16^), 1.85 for FPS (95% CI, 1.56−2.20; *P* = 8.3 × 10^−13^), and 1.6 for PPS (95% CI, 1.30−1.98; *P* = 8.3 × 10^−6^). High *AURKB* expression levels were associated with a two- to three-fold reduction in survival among patients, indicating a poor prognosis for lung cancer. High expression levels of *BIRC5*, *CDCA8*, and *INCENP*, the co-components of CPC, were also significantly associated with low survival rates in patients. Furthermore, high expression of these genes significantly affected the FPS, referring to the time from treatment initiation to the first progression of the disease, in lung adenocarcinoma. As shown in ***Table 1*** and ***Supplementary Fig. 2*** (available online), high expression levels of *AURKB* (HR = 2.14; 95% CI, 1.74−2.63; *P* = 9.0 × 10^−14^), *BIRC5* (HR = 2.45; 95% CI, 1.99−3.02; *P* < 1.0 × 10^−16^), *CDCA8* (HR = 1.79; 95% CI, 1.46−2.20; *P* = 9.6 × 10^−9^), and *INCENP* (HR = 1.89; 95% CI, 1.55−2.32; *P* = 3.2 × 10^−10^) were associated with poor FPS in LUAD cases, but not in LUSC cases. These findings suggest that CPC components may be considered prognostic biomarkers for LUAD.

**Table 1:** First progression survival analysis across subtypes of non-small cell lung cancer

CPC genes	Lung adenocarcinoma						Lung squamous cell carcinoma				
	Patient (*n*)	HR (95% CI)	*P*	Low (months)	High (months)		Patient (*n*)	HR (95% CI)	*P*	Low (months)	High (months)
*AURKB*	906	2.14 (1.74−2.63)	9.0×10^−14^	35.00	11.00		220	0.90 (0.60−1.36)	6.3×10^−1^	13.33	11.00
*BIRC5*	906	2.45 (1.99−3.02)	<1.0×10^−16^	40.00	11.00		220	0.92 (0.61−1.39)	7.0×10^−1^	68.00	104.00
*CDCA8*	906	1.79 (1.46−2.20)	9.6×10^−9^	31.61	12.00		220	1.03 (0.69−1.55)	8.7×10^−1^	12.55	13.00
*INCENP*	906	1.89 (1.55−2.32)	3.2×10^−10^	164.00	43.00		220	0.91 (0.61−1.37)	6.5×10^−1^	11.17	14.00
The mRNA levels of CPC genes were classified into low and high expression groups according to the median expression of each gene. First progression survival is shown as median survival time in months. Abbreviations: CI, confidence interval; HR, hazard ratio; OR, odds ratio.											

Angiogenesis, hypoxia, and immune cell infiltration in the LUAD tumor microenvironment may lead to the overexpression of CPC, promoting aggressive tumor growth. The dysregulation of CPC components may result in aberrant cell growth and proliferation, leading to cancer development. To prevent these processes, checkpoints, CDKs, and cyclins regulate the proper cell cycle. Therefore, the current study also analyzed the correlation between CPC molecules and cell cycle checkpoints using the ENCORI database^[7]^. The results showed that expression levels of *AURKB*, *BIRC5*, *CDCA8*, and *INCENP* were highly correlated with those of the cyclin E/CDK2 complex (*CCNE1* and *CDK2*) and cyclin B/CDK1 complex (*CCNB1* and *CDK1*), indicating their significant roles during the S and M phases of the cell cycle (***Table 2***; ***Supplementary Figs. 3*** and ***4***, available online). The strong correlation between CPC and cyclin E/CDK2, along with cyclin B/CDK1, suggests that, under normal conditions, CPC components ensure proper assembly and function of the mitotic spindle and accurate DNA replication in the S phase before mitosis begins. In the M phase, CPC is critical for the metaphase-to-anaphase transition, ensuring proper chromosomal segregation. The dysregulation of these processes, resulting from the overexpression of CPC and cyclin/CDKs, may lead to enhanced cell proliferation and tumor aggressiveness. These findings suggest that the dysregulation of the CPC can promote tumor growth and highlight the need for close monitoring of these checkpoints, as any dysfunction in their control molecules may accelerate cancer progression.

**Table 2:** Correlation analysis between CPC and cyclin/CDKs in lung adenocarcinoma

CPC genes	*CCND1*			*CCNE1*			*CCNA1*			*CCNB1*			*CDK4*			*CDK6*			*CDK2*			*CDK1*	
	*r*	*P*		*r*	*P*		*r*	*P*		*r*	*P*		*r*	*P*		*r*	*P*		*r*	*P*		*r*	*P*
*AURKB*	−0.13	1.41×10^−3^		0.66	4.99×10^−68^		−0.02	5.91×10^−1^		0.85	3.75×10^−154^		0.49	3.48×10^−34^		0.13	1.39×10^−3^		0.67	5.06×10^−72^		0.81	1.19×10^−127^
*BIRC5*	−0.14	1.11×10^−3^		0.69	6.33×10^−77^		<0.01	8.75×10^−1^		0.87	3.17×10^−169^		0.52	1.33×10^−38^		0.12	2.97×10^−3^		0.66	1.45×10^−68^		0.87	4.03×10^−167^
*CDCA8*	−0.13	1.45×10^−3^		0.71	1.29×10^−82^		0.01	7.39×10^−1^		0.84	1.88×10^−143^		0.46	2.75×10^−29^		0.22	2.45×10^−7^		0.72	2.43×10^−85^		0.83	2.33×10^−139^
*INCENP*	−0.01	8.21×10^−1^		0.55	2.09×10^−44^		0.00	8.82×10^−1^		0.61	1.25×10^−56^		0.32	3.97×10^−14^		0.33	4.80×10^−15^		0.63	6.31×10^−61^		0.60	1.76×10^−53^
Abbreviations: CDKs, cyclin-dependent kinases; CPC, chromosomal passenger complex.																							

A weak correlation was found between the mRNA levels of the CPC genes and those of the proteins related to the G0/G1 checkpoint, while no correlation was observed between the CPC genes' mRNA levels and those of the proteins associated with the G2 checkpoint, indicating that the CPC may bypass normal checks and balances in LUAD, possibly enhancing tumor aggressiveness. The G0/G1 phase is primarily involved in growth and DNA replication, while the G2 phase is critical for cell maturation and mitosis. A missed checkpoint may lead to aneuploidy, chromosomal instability, or mutations, thereby resulting in rapid tumor growth, metastasis, and a poor prognosis. During the G0 phase, which represents cell cycle arrest or quiescence, stable CPC components may be targeted to reduce the risk of recurrence.

Using the KM Plotter database, we subsequently revealed a strong association between cyclins and CDKs, which are involved in the S and M phases of the cell cycle, and the PFS in lung cancer patients (***Supplementary Fig. 5*** and ***Supplementary Table 2***, available online). The analysis further demonstrated a hazard ratio of 1.8 to > 2 in LUAD, as shown in ***Supplementary Fig. 6*** (available online) and ***Table 3***. These findings suggest that cyclins and CDKs may serve as significant predictive biomarkers for the poor prognosis of LUAD.

**Table 3:** First progression survival analysis of checkpoints in NSCLC subtypes

Cyclin/CDKs	Lung adenocarcinoma						Lung squamous cell carcinoma				
	Patient (*n*)	HR (95% CI)	*P*	Low (months)	High (months)		Patient (*n*)	HR (95% CI)	*P*	Low (months)	High (months)
S phase											
*CCNE1*	906	2.16 (1.76−2.66)	4.5×10^−14^	35.00	11.00		220	1.37 (0.91−2.06)	1.3×10^−1^	14.00	11.17
*CDK2*	906	2.41 (1.96−2.97)	<1.0×10^−16^	34.90	11.00		220	1.16 (0.78−1.75)	4.6×10^−1^	13.01	12.13
M phase											
*CCNB1*	906	2.21 (1.62−3.01)	2.4×10^−7^	69.00	17.93		220	1.31 (0.87−1.97)	1.9×10^−1^	18.33	11.00
*CDK1*	906	2.38 (1.94−2.93)	<1.0×10^−16^	40.00	10.00		220	1.07 (0.71−1.61)	7.4×10^−1^	14.00	11.83
The mRNA levels of cyclin/CDK genes were classified into low and high expression groups according to the median expression of each gene. First progression survival is shown as median survival time in months. Abbreviations: CI, confidence interval; HR, hazard ratio; NSCLC, non-small cell lung cancer; OR, odds ratio.											

The current study is the first to establish the correlation between CPC and cell cycle checkpoints and their prognostic significance in lung cancer. Given the high hazard ratios for FPS, patients with elevated CPC-cyclin/CDK expression are more likely to experience recurrence and progression. Therefore, targeting both CPC and cyclin/CDKs in LUAD patients may potentially improve treatment outcomes and reduce recurrence. ***Fig. 1*** illustrates our hypothesis regarding how the CPC and cyclin/CDKs dysregulate the lung cancer cell cycle compared with normal lung cells. However, future research is needed to validate this hypothesis and to elucidate the regulatory mechanisms. Prospective longitudinal studies may enhance the effectiveness and safety of targeted therapies by using a multi-targeted approach. This approach addresses multiple disease progression pathways, thereby reducing the risk of resistance and improving overall patient outcomes.

**Figure 1:**
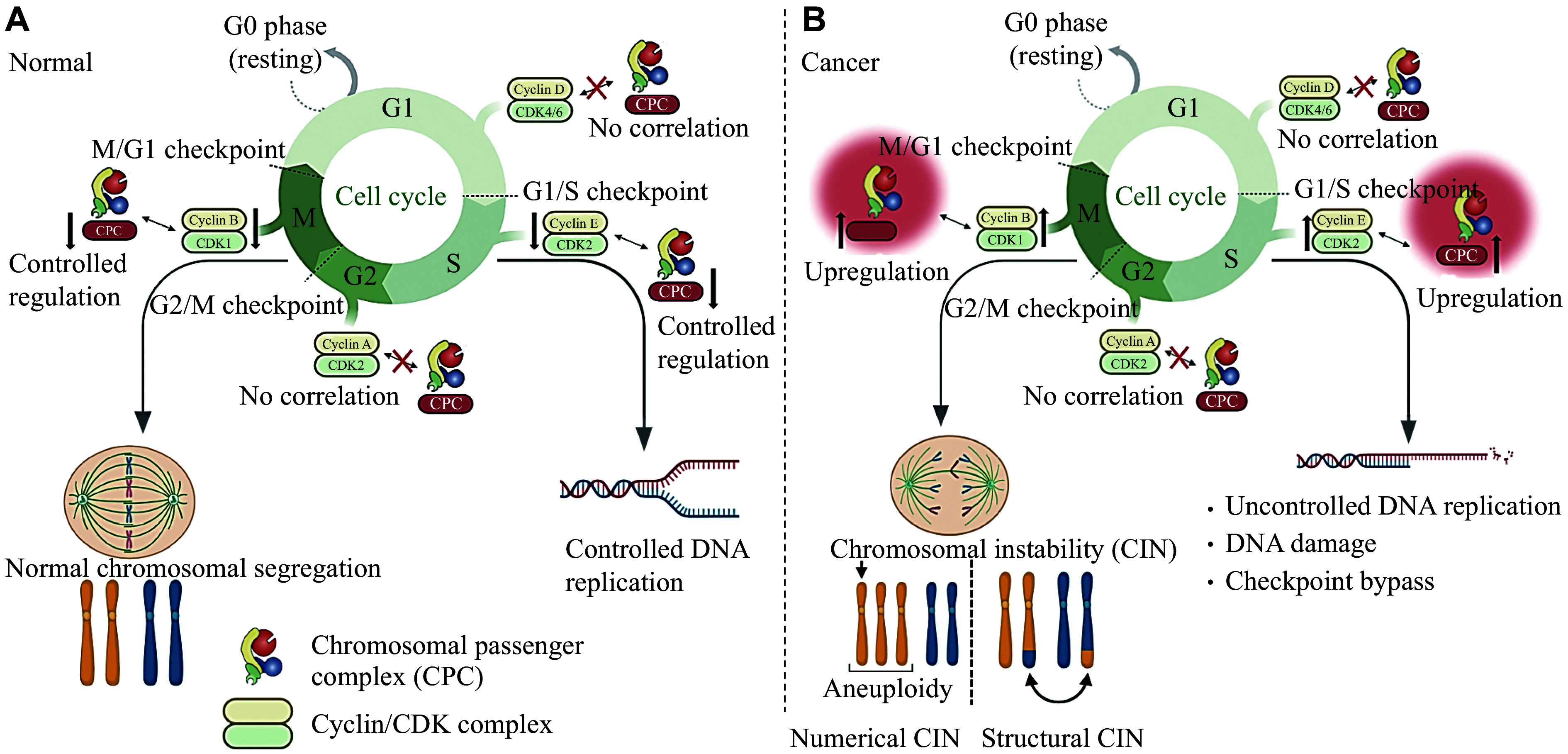
Correlations between chromosomal passenger complex (CPC) and cell cycle checkpoints in different phases of the cell cycle. A: Normal condition characterized by regulated cell cycle progression and controlled expression of CPC, cyclin E/CDK2, and cyclin B/CDK1. B: Cancerous condition exhibits an upregulation of the S phase (cyclin E/CDK2) and M phase (cyclin B/CDK1) checkpoints, coupled with overexpression of CPC genes. As a result of this dysregulation, excessive DNA replication, checkpoint bypass, improper chromosomal segregation (chromosomal instability [CIN]), and aneuploidy occur.

We would like to thank the funding support through Manipal University Jaipur for the Enhanced Seed Grant under the Endowment Fund (Grant No. E3/2023-24/QE-04-05).

Yours sincerely,Prerna Vats^1^, Sakshi Nirmal^1^, Ashok Kumar^2^, Rajeev Nema^1,✉^ ^1^Department of Biosciences, Manipal University Jaipur, Jaipur, Rajasthan 303007, India;^2^Department of Biochemistry, All India Institute of Medical Sciences (AIIMS),Bhopal, Madhya Pradesh 462020, India.^✉^Corresponding author: Rajeev Nema. E-mail: rajeev.nema@jaipur.manipal.edu.

## SUPPLEMENTARY DATA

Supplementary data to this article can be found online.
