# Supplementary material for: Chromosomal passenger complex-cyclin/CDK axis correlated with poor lung cancer prognosis
Source: J Biomed Res. 2025 Feb 8;39(5):530–3. doi: 10.7555/JBR.38.20240296 (PMC12481672; doi:10.7555/JBR.38.20240296)
Supplement: Supplementary file 1 — Supplementary data to this article can be found online. [file jbr-39-5-530-Supplementary.pdf]

Available online at www.jbr-pub.org.cn

Open Access at PubMed Central

JBR

Journal of Biomedical Research, 2025 39(5): 1–7

Supplementary Data

Chromosomal passenger complex-cyclin/CDK axis correlated with poor lung cancer prognosis

Prerna Vats[1], Sakshi Nirmal[1], Ashok Kumar[2], Rajeev Nema[1,✉]

[1]*Department of Biosciences, Manipal University Jaipur, Jaipur, Rajasthan 303007, India;*
[2]*Department of Biochemistry, All India Institute ofMedical Sciences (AIIMS), Bhopal, Madhya Pradesh 462020, India.*

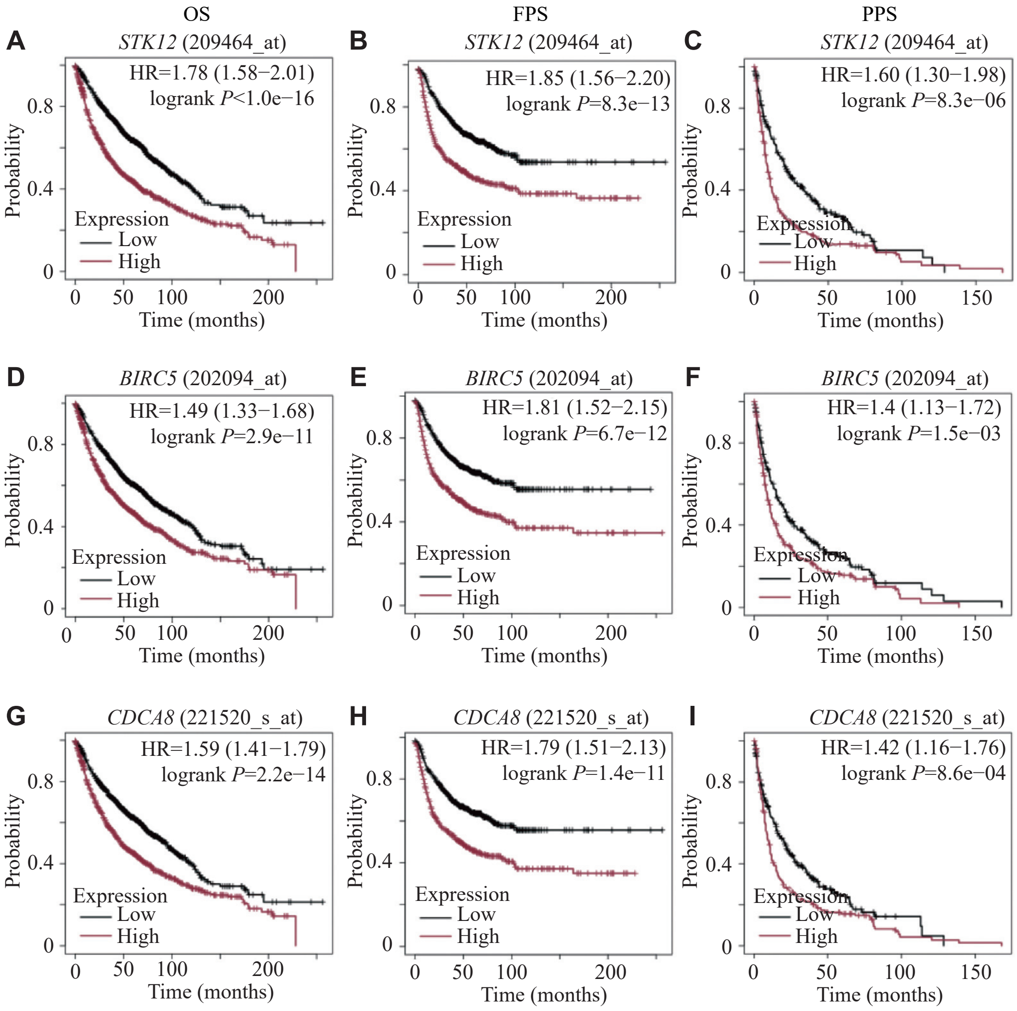

(Continued)

✉Corresponding author: Rajeev Nema, Department of Biosciences, Manipal University Jaipur, Dehmi Kalan, Jaipur-Ajmer Expressway, Jaipur, Rajasthan 303007, India. E-mail: rajeev.nema@jaipur.manipal.edu.

Received: 11 September 2024; Revised: 31 December 2024; Accepted: 07 January 2025; Published online: 08 February 2025

CLC number: R734.2, Document code: B

The authors reported no conflict of interests.

This is an open access article under the Creative Commons Attribution (CC BY 4.0) license, which permits others to distribute, remix, adapt and build upon this work, for commercial use, provided the original work is properly cited.

© 2025 by Journal of Biomedical Research.

https://doi.org/10.7555/JBR.38.20240296

(Continued)

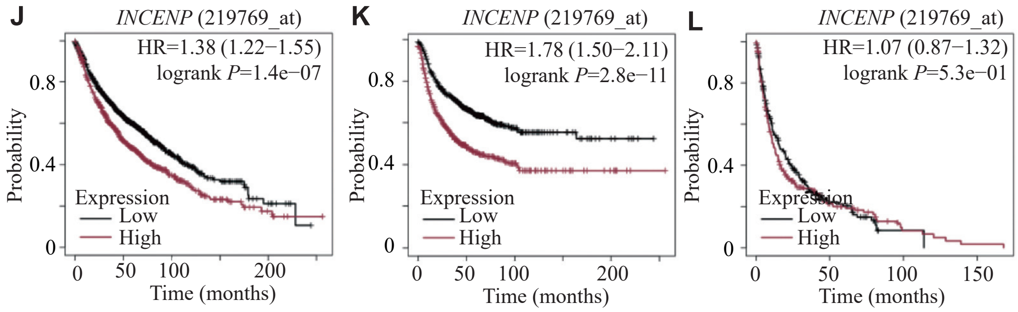

Supplementary Fig. 1 **Survival analysis of the chromosomal passenger complex (CPC) in lung cancer patients.** A–L: Overall survival (OS; total *N* = 2 166: low, *n* = 1085; high, *n* = 1081), first progression survival (FPS; *N* = 1 252: low, *n* = 627; high, *n* = 625), and post-progression survival (PPS; *N* = 477: low, *n* = 238; high, *n* = 239) in lung cancer patients were analyzed using the KM Plotter for *AURKB* (*STK12*; A–C), *BIRC*5 (D–F), *CDCA8* (G–I), and *INCENP* (J–L), respectively. The mRNA levels of CPC genes were classified into low and high expression groups according to the median expression of each gene.

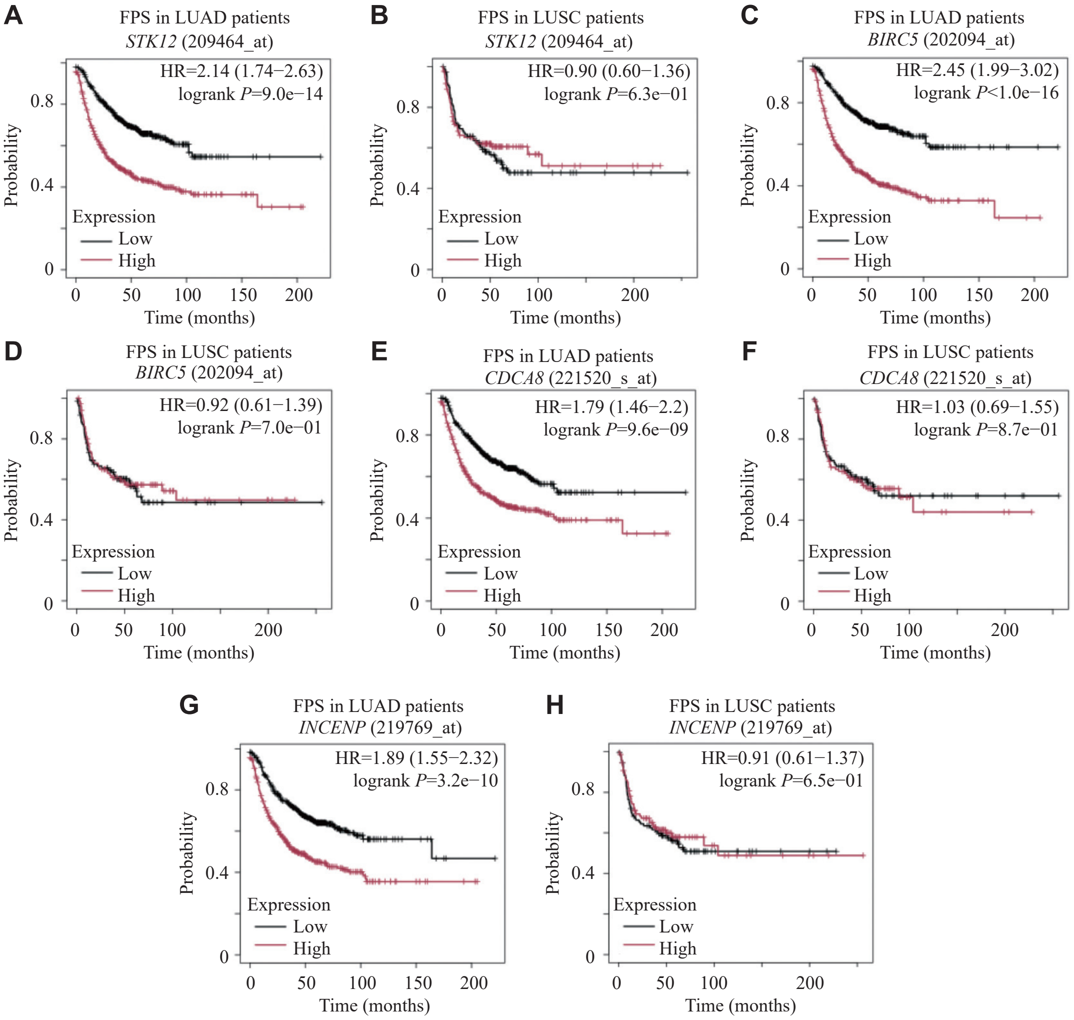

Supplementary Fig. 2 **Survival analysis of the chromosomal passenger complex (CPC) in lung adenocarcinoma (LUAD) and lung squamous cell carcinoma (LUSC) patients.** A–H: First progression survival (FPS) in LUAD (*N* = 906: low, *n* = 453; high, *n* = 453) and LUSC (*N* = 220: low, *n* = 110; high, *n* = 110) patients was analyzed using the KM Plotter for *AURKB* (*STK12*; A and B), *BIRC5* (C and D), *CDCA*8 (E and F), and *INCENP* (G and H), respectively. The mRNA levels of CPC genes were classified into low and high expression groups according to the median expression of each gene.

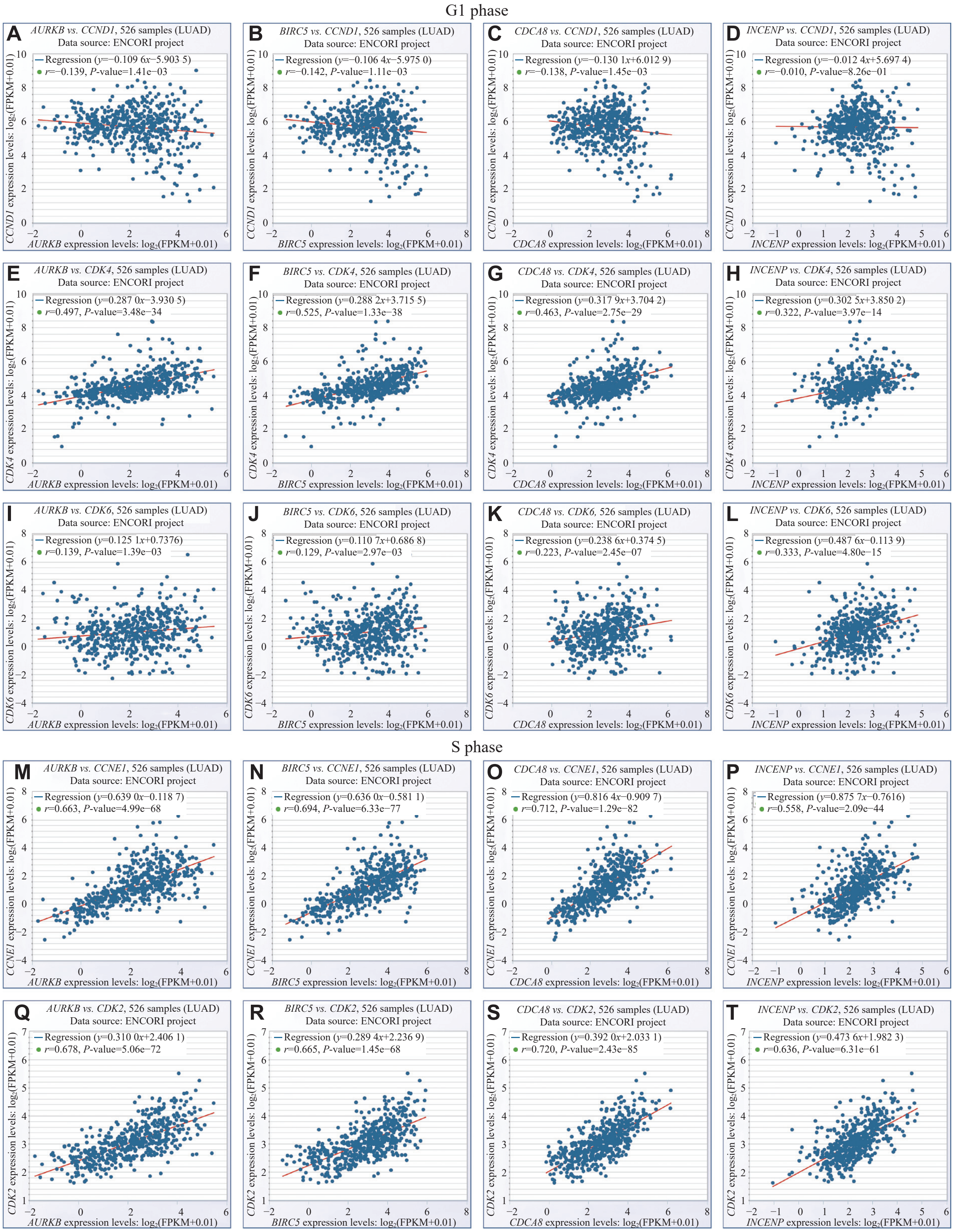

Supplementary Fig. 3 **Correlation analysis between chromosomal passenger complex (CPC) molecules and cell cycle checkpoints (G0/G1 and S phases).** Correlation analysis between the mRNA levels of CPC genes and those of the cyclin/CDK of the G0/G1 phase (*CCND1*/*CDK4* and *CCND1*/*CDK6*; A–L) and the S phase (*CCNE1*/*CDK2*; M–T) in lung adenocarcinoma patients (n = 526) using the ENCORI database.

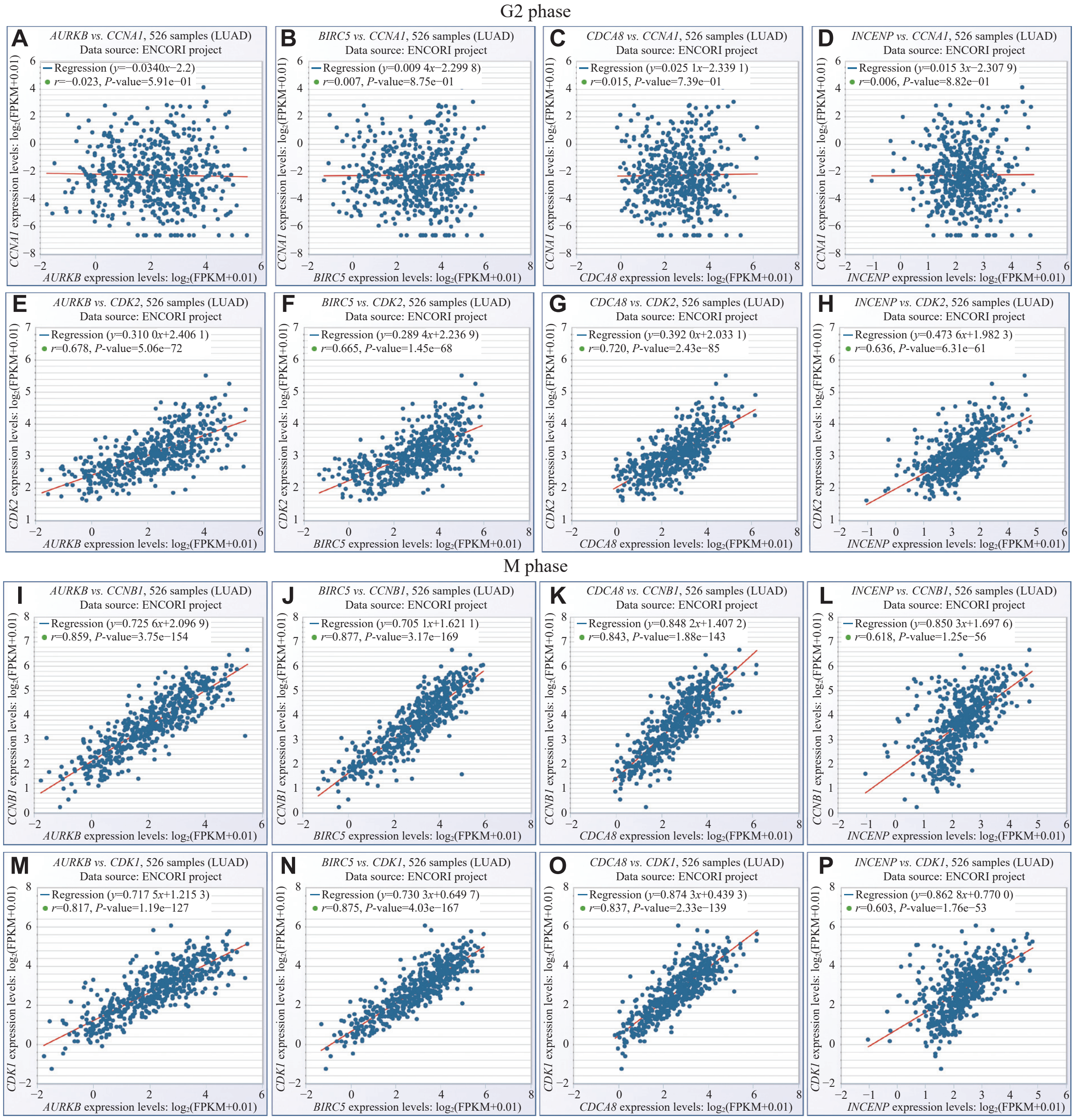

Supplementary Fig. 4 **Correlation analysis between chromosomal passenger complex (CPC) molecules and cell cycle checkpoints (G2 and M phases).** Correlation analysis between the mRNA levels of CPC genes and those of the cyclin/CDK of the G2 phase (*CCNA1*/*CDK2*; A–H) and the M phase (*CCNB1*/*CDK1*; I–P) in lung adenocarcinoma patients (n = 526) using the ENCORI database.

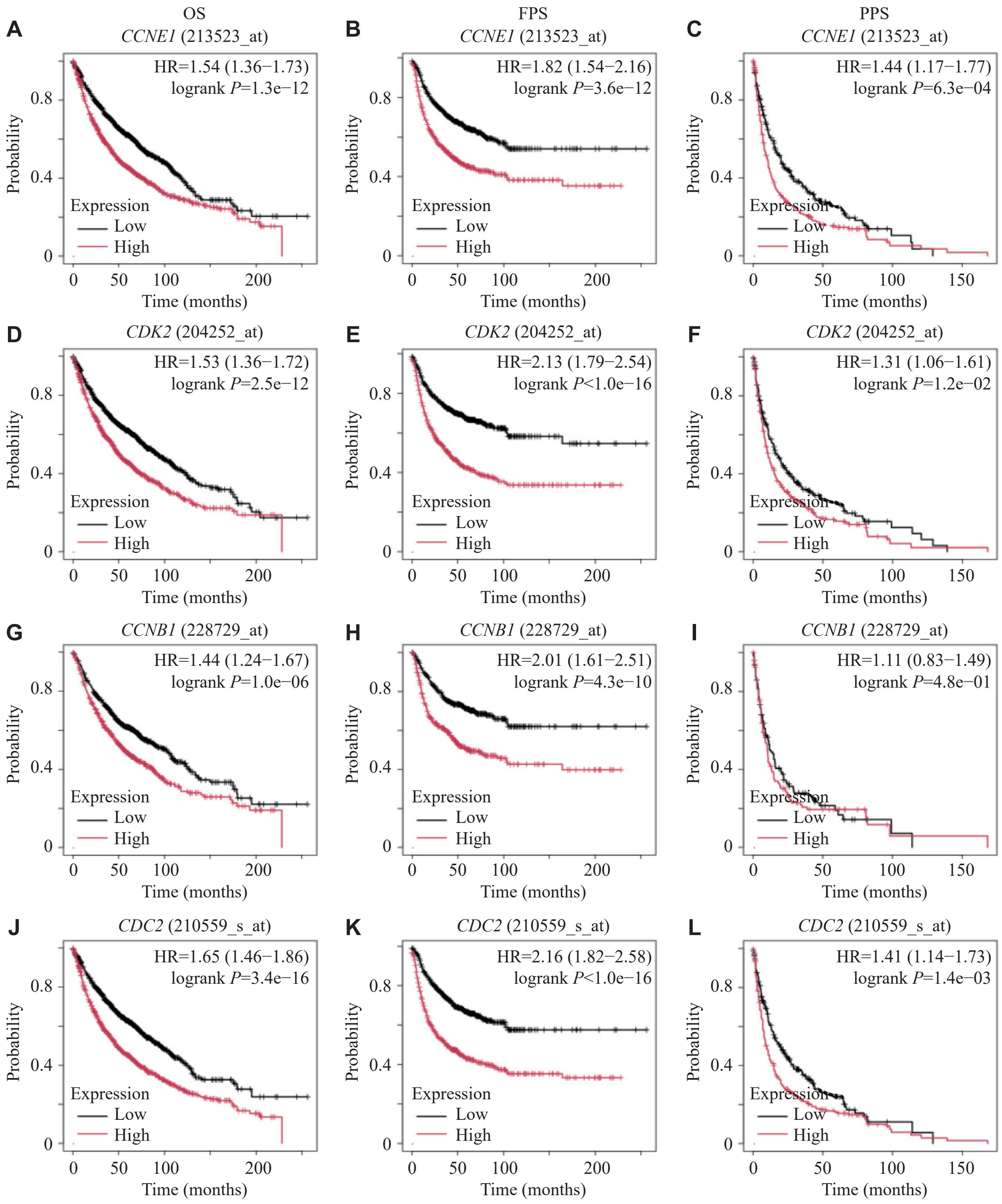

Supplementary Fig. 5 **Survival analysis of S phase and M phase checkpoints in lung cancer patients.** Overall survival (OS; total $N = 2\ 166$: low, $n = 1\ 084$; high, $n = 1\ 082$), first progression survival (FPS; $N = 1\ 252$: low, $n = 627$; high, $n = 625$), and post-progression survival (PPS; $N = 477$: low, $n = 238$; high, $n = 239$) analysis of S phase (*CCNE1*/*CDK2*; A–F) and M phase (*CCNB1*/*CDC2*; G–L) checkpoints in lung cancer patients using the KM Plotter. The mRNA levels of cyclin/CDK genes were classified into low and high expression groups according to the median expression of each gene.

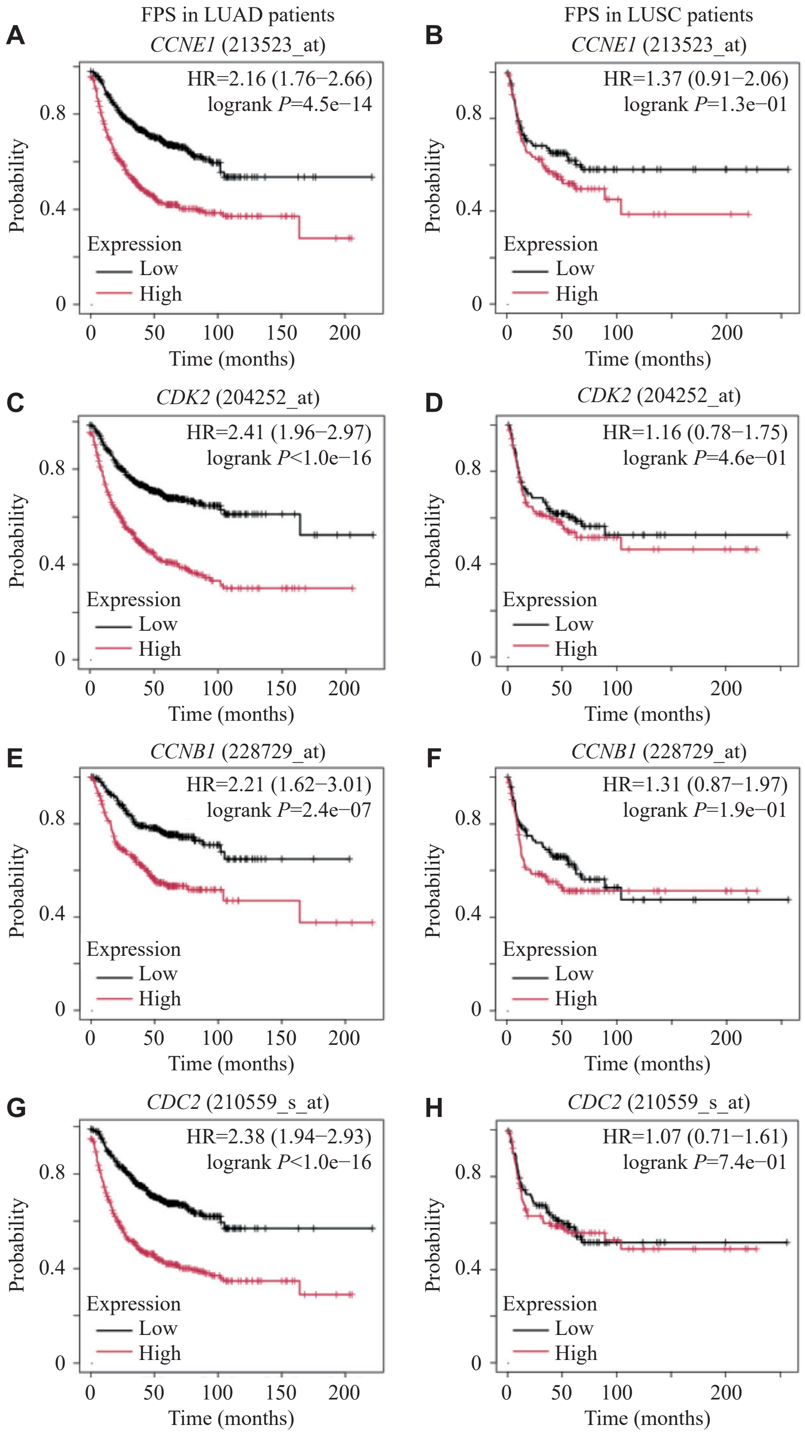

Supplementary Fig. 6 **Survival analysis of S phase and M phase checkpoints in lung adenocarcinoma (LUAD) and squamous cell carcinoma (LUSC) patients.** A–H: First progression survival (FPS) of S phase (*CCNE1*/*CKD2*; A–D) and M phase (*CCNB1*/*CDC2*; E–H) checkpoints in LUAD (N = 906: low, n = 453; high, n = 453) and LUSC (N = 220; low, n = 110; high, n = 110) patients were analyzed using the KM Plotter. The mRNA levels of cyclin/CDK genes were classified into low and high expression groups according to the median expression of each gene.

Supplementary Table 1 **Prognostic significance of chromosomal passenger complex components in lung cancer patients**

CPC genes	Overall survival					First progression survival					Post progression survival				
	Patient (*n*)	HR (95% CI)	*P*	Low (months)	High (months)	Patient (*n*)	HR (95% CI)	*P*	Low (months)	High (months)	Patient (*n*)	HR (95% CI)	*P*	Low (months)	High (months)
AURKB	2 166	1.78 (1.58−2.01)	<1.0e−16	93.00	43.83	1 252	1.85 (1.56−2.20)	8.3e−13	31.61	10.00	477	1.60 (1.30−1.98)	8.3e−06	21.90	9.00
BIRC5	2 166	1.49 (1.33−1.68)	2.9e−11	86.00	51.27	1 252	1.81 (1.52−2.15)	6.7e−12	28.70	11.00	477	1.40 (1.13−1.72)	1.5e−03	19.47	10.38
CDCA8	2 166	1.59 (1.41−1.79)	2.2e−14	94.50	48.00	1 252	1.79 (1.51−2.13)	1.4e−11	29.00	11.00	477	1.42 (1.16−1.76)	8.6e−04	21.00	10.00
INCENP	2 166	1.38 (1.22−1.55)	1.4e−07	86.00	54.00	1 252	1.78 (1.50−2.11)	2.8e−11	27.00	11.00	477	1.07 (0.87−1.32)	5.3e−01	15.62	12.00

The mRNA levels of CPC genes were classified into low and high expression groups according to the median expression of each gene. First progression survival is shown as median survival time in months.
Abbreviations: HR, hazard ratio; OR, odds ratio; CI, confidence interval.

Supplementary Table 2 **Prognostic significance of S and M phase cell cycle checkpoints**

Cyclin/ CDKs	Overall survival					First progression survival					Post progression survival				
	Patient (*n*)	HR (95% CI)	*P*	Low (months)	High (months)	Patient (*n*)	HR (95% CI)	*P*	Low (months)	High (months)	Patient (*n*)	HR (95% CI)	*P*	Low (months)	High (months)
S phase															
CCNE1	2 166	1.54 (1.36−1.73)	1.3e−12	93.00	48.60	1 252	1.82 (1.54−2.16)	3.6e−12	29.00	11.00	477	1.44 (1.17−1.77)	6.3e−04	20.00	9.00
CDK2	2 166	1.53 (1.36−1.72)	2.5e−12	91.00	49.00	1 252	2.13 (1.79−2.54)	<1.0e−1 6	32.57	11.00	477	1.31 (1.06−1.61)	1.2e−02	17.25	10.78
M phase															
CCNB1	2 166	1.44 (1.24−1.67)	1.0e−06	102.00	56.50	1 252	2.01 (1.61−2.51)	4.3e−10	40.00	13.00	477	1.11 (0.83−1.49)	4.8e−01	12.00	10.38
CDK1	2 166	1.65 (1.46−1.86)	3.4e−16	95.50	48.00	1 252	2.16 (1.82−2.58)	<1.0e−1 6	35.00	10.00	477	1.41 (1.14−1.73)	1.4e−03	19.50	10.00

The mRNA levels of cyclin/CDK genes were classified into low and high expression groups according to the median expression of each gene. First progression survival is shown as median survival time in months.
Abbreviations: HR, hazard ratio; OR, odds ratio; CI, confidence interval.
